# Metformin Alleviates Neuroinflammation Following Intracerebral Hemorrhage in Mice by Regulating Microglia/Macrophage Phenotype in a Gut Microbiota-Dependent Manner

**DOI:** 10.3389/fncel.2021.789471

**Published:** 2022-01-18

**Authors:** Xiaobo Yu, Xiongjie Fu, Xinyan Wu, Wenwen Tang, Lei Xu, Libin Hu, Chaoran Xu, Hang Zhou, Guoyang Zhou, Jianru Li, Shenglong Cao, Jiang Liu, Feng Yan, Lin Wang, Fuyi Liu, Gao Chen

**Affiliations:** ^1^Department of Neurosurgery, Second Affiliated Hospital, School of Medicine, Zhejiang University, Hangzhou, China; ^2^School of Medicine, Zhejiang University, Hangzhou, China; ^3^Department of Neurosurgery, China-Japan Friendship Hospital, Beijing, China

**Keywords:** intracerebral hemorrhage, metformin, neuroinflammation, gut microbiota, microglia/macrophage

## Abstract

The gut microbiota plays a key role in regulating intracerebral hemorrhage (ICH)-induced neuroinflammation. The anti-neuroinflammatory effects of metformin (Met) have been reported in many central nervous system (CNS) diseases. However, whether Met regulates neuroinflammation through the gut microbiota in ICH-induced brain injury remains unknown. We found that Met treatment substantially alleviated neurological dysfunction and reduced neuroinflammation by inhibiting pro-inflammatory polarization of microglia/macrophages in mice with ICH. Moreover, Met treatment altered the microbiota composition and improved intestinal barrier function. The expression of lipopolysaccharide-binding protein (LBP), a biomarker of intestinal barrier damage, was also significantly reduced by Met treatment. Neuroinflammation was also potently ameliorated after the transplantation of fecal microbiota from Met-treated ICH mice. The neuroprotective effects of fecal microbiota transplantation (FMT) were similar to those of oral Met treatment. However, suppression of the gut microbiota negated the neuroprotective effects of Met in ICH mice. Therefore, Met is a promising therapeutic agent for neuroinflammation owing to ICH-induced imbalance of the gut microbiota.

## Introduction

Intracerebral hemorrhage (ICH) is a common endovascular disease mainly related to aging and hypertension and shows poor outcomes and high mortality rates (Taylor et al., 2017; Zhu et al., 2019). ICH causes acute brain injury, which can lead to primary and secondary injuries (Zhu et al., 2019). Primary brain injury is induced by the hematoma and hematoma growth, whereas secondary brain injury involves neuroinflammation, cytotoxicity, oxidative stress, white matter injury, and neuronal apoptosis (Zhao et al., 2014; Duan et al., 2016; Li et al., 2017; Fu et al., 2021b, c). Surgical hematoma evacuation has been associated with protective effects such as the alleviation of mass effects and hematoma-related brain injury. However, this method fails to improve neurological function in patients with ICH (Mendelow et al., 2013). Therefore, a new therapeutic strategy for ICH is urgently needed.

Microglia/macrophage-mediated neuroinflammatory responses following ICH are closely associated with prognosis in patients with ICH. Previously, studies on ICH-induced neuroinflammation focused on cellular receptors, pharmacology, and genetics (Chang et al., 2020, 2021; Lu et al., 2021). However, recent research has revealed that the gut microbiota is involved in acute central nervous system (CNS) injury (Benakis et al., 2016; Celorrio et al., 2021; Jing et al., 2021). The intestinal tract contains a variety of microorganisms which influence the host body’s physiology (Agirman and Hsiao, 2021). The gut microbiota is involved in many CNS diseases, such as ischemic stroke (IS), spinal cord injury (SCI), traumatic brain injury (TBI), and ICH through the “gut-brain axis” (Benakis et al., 2016; Sadler et al., 2020; Celorrio et al., 2021; Jing et al., 2021; Yu et al., 2021; Zhang et al., 2021). The regulation of the gut microbiota has been reported to influence inflammation and disease outcomes in mice with IS. Gut microbiota-derived short-chain fatty acids have been shown to improve locomotor and gastrointestinal functions and neuroinflammation in mice with SCI. In addition, TBI-induced gut microbiota dysbiosis affects neuroinflammation, neurogenesis, and fear memory. Our previous study showed that ICH can lead to an imbalance in the gut microbiota and aggravate neuroinflammation.

Metformin (Met) is a drug widely used to treat metabolic diseases (He and Wondisford, 2015; Clarke, 2021). It has potent anti-inflammation and immuno-suppressive effects (Ou et al., 2018). Recently, numerous studies demonstrated that Met exerts neuroprotective effects in a gut microbiota-dependent manner (Ma et al., 2021). Moreover, Met was reported to attenuate ICH-induced neurological deficits (Qi et al., 2017). However, whether the regulation of the gut microbiota mediates the neuroprotective effects of Met following ICH, remains unknown.

In this study, we investigated the effect of Met on ICH-induced neuroinflammation, the changes in the microglial/macrophage phenotype, and the functional changes in the gastrointestinal tract. We found that Met exerted anti-inflammatory effects, promoted neurofunctional recovery, and improved gut barrier function following ICH. Administration of Met-treated feces had neuroprotective effects and improved the neurobehavioral function in mice with ICH. However, treatment with broad-spectrum antibiotics reversed the protective effects of Met following ICH. These results indicate that the neuroprotective effects of Met depend on the gut microbiota. Our data indicate the role of the gut-brain connection in ICH treatment and may that it may contribute to the development of therapeutic strategies for ICH.

## Materials and Methods

### Animals

C57 mice were purchased from Charles River Laboratory Animal Co., Ltd. (Beijing, China). In total, 298 adult male mice (6–8 weeks old, 22–25 g) were used in this study. Mice were housed under specific pathogen-free (SPF) conditions in a temperature- and humidity-controlled room, under a standard 12-h light/dark cycle with free access to food and water. All procedures were conducted according to the Guidelines for the Care and Use of Laboratory Animals published by the National Institutes of Health. All procedures were approved and supervised by the Institutional Ethics Committee of the Second Affiliated Hospital, Medicine School of Zhejiang University. The study groups and number of animals per group are shown in Supplementary Table 1.

### Study Design

#### Experiment 1

To evaluate the role of Met following ICH, 20 mice were randomly divided into the following two groups, i.e., ICH + vehicle (*n* = 10) and ICH + Met (*n* = 10). The ICH + vehicle group was treated with the vehicle used for Met following the ICH. The ICH + Met group was treated with Met following the induction of ICH. The body weight and neurological scores of the mice in each group were recorded at baseline and at 1, 3, and 7 days post ICH induction.

#### Experiment 2

To investigate the effect of Met on ICH-induced neuroinflammation, 38 mice were randomly divided into six groups [sham d3 (*n* = 3), sham d7 (*n* = 3), ICH d3 + vehicle (*n* = 8), ICH d7 + vehicle (*n* = 8), ICH d3 + Met (*n* = 8), and ICH d7 + Met (*n* = 8)] and analyzed by quantitative real-time polymerase chain reaction (qRT-PCR) and immunofluorescence staining. The polarization status of the microglia/macrophages in the six groups at 3 and 7 days post ICH induction with and without Met treatment was analyzed using immunofluorescence staining (*n* = 5 per group) and qRT-PCR (*n* = 3 per group).

#### Experiment 3

To examine the effects of Met on gut function impairment after ICH, 90 mice were randomly divided into five groups [sham d3 (*n* = 15), sham d7 (*n* = 15), ICH d3 + vehicle (*n* = 15), ICH d7 + vehicle (*n* = 15), ICH d3 + Met (*n* = 15), and ICH d7 + Met (*n* = 15)] and analyzed with gut permeability test, enzyme-linked immunosorbent assay (ELISA), and immunofluorescence staining. Plasma fluorescence measurements were used to analyze the gut permeability (*n* = 4 per group). Immunofluorescence staining (*n* = 5 per group) and lipopolysaccharide (LPS)-binding protein (LBP) assay (*n* = 6 per group) were performed to analyze the intestinal barrier function at days 3 and 7 post ICH induction with and without Met treatment.

#### Experiment 4

To study the changes in the gut microbiome of mice with ICH after Met treatment, 10 mice were randomly divided into either the ICH + vehicle or ICH + Met groups (*n* = 5 per group) Fecal matter was collected preoperatively and after ICH induction (3 days), and after ICH induction and treatment with Met (3 days). 16S rRNA gene sequencing was then performed.

#### Experiment 5

To confirm the key role of the gut microbiota in mediating the neuroprotective effects of Met, antibiotic treatments were performed. Thirty-four mice were randomly divided into three groups: sham (*n* = 6), ICH + Met (*n* = 14), and ICH + Met with antibiotic treatment (*n* = 14) groups. Before ICH induction, mice in the antibiotic treatment group mice were treated with freshly made broad-spectrum antibiotics added in the drinking water daily for 4 weeks. After ICH, two groups of mice were administered with Met and then subjected to neurobehavioral assessment, immunofluorescence staining, and qRT-PCR analysis.

#### Experiment 6

FMT experiments were performed to evaluate the mechanism underlying the neuroprotective effects of Met. Seventy-nine mice were randomly divided into three groups [sham (*n* = 13), ICH + AMNV + FMT (vehicle; *n* = 33), and ICH + AMNV + FMT (Met; *n* = 33)] and analyzed based on neurobehavioral assessment, immunofluorescence staining, qRT-PCR, gut permeability test, and ELISA.

### ICH Mouse Model

ICH mouse models were established as previously described to simulate the series of pathophysiological changes after the onset of ICH (Chang et al., 2018). Briefly, the mice were anesthetized intraperitoneally with pentobarbital sodium (40mg/kg, 1%). Then, 0.05 U collagenase (type VII, from Clostrid-iumhistolyticum; Sigma-Aldrich) in 0.5 μl saline which was prepared within 1 h of usage and kept in an ice bath was stereotactically injected into the right basal ganglia region (2.5 mm lateral to the bregma, 3 mm deep at a 5° angle) over 5 min, followed by another 5 min of observation to detect the reflux. The rectal temperature was reported throughout the ICH induction and maintained at 37.0°C ± 0.5°C. Mice in the sham group received the same treatment, but without collagenase during needle insertion.

### Met Administration

Met (Merck Serono, Switzerland) was dissolved in 0.9% saline to prepare a solution with a concentration of 0.25 g ml^−1^. The dose of Met was determined as described previously (Hill et al., 2016). Mice in the Met group were treated with Met with a dose of 250 mg kg^−1^ d^−1^ by oral gavage, 30 min after ICH induction and then daily for 7 days, whereas mice in the vehicle group were treated with an equal volume of saline.

### Assessment of Neurobehavioral Function

Two researchers who were blinded to the experiments assessed the neurobehavioral function of the mice. The cylinder test, forelimb placing test, and wire hanging tests were conducted to evaluate the neurobehavioral function on days 1, 3, and 7 after ICH onset (Li et al., 2020; Fu et al., 2021a; Yang et al., 2021). In the cylinder test, the mice were placed in a plastic cylinder of 8 cm in diameter and 25 cm in height, which allowed the mice free rearing up to 20 times. The researchers recorded the location of the first forelimb on the wall, and the score was calculated as follows: (right − left)/(right + left + both). A greater positive score suggested a more severe left hemiparesis. In the forelimb placing test, the torso of each mouse was held and the forelimb was allowed to hang freely. For every mouse, each forelimb was tested 10 times. The percentage of the number of times that the mice placed the appropriate forelimb on the edge of the countertop in response to vibrissae stimulation was determined. For the wire-hanging test, the mice were placed on a stainless steel bar (50 cm in length and 2 mm in diameter), which rested on two vertical supports and fixed at a height of 37 cm above a flat table. The mice were tested for 30 s in three consequent trials. The scores were calculated as following: 0 (fell off), 1 (held the bar with two forepaws), 2 (held the bar with an additional attempt to climb onto the bar), 3 (held the bar with two forepaws and one or two hind paws), 4 (held the bar with all four paws and with the tail wrapped around the bar), and 5 (escaped to one of the supports).

### qRT-PCR

Total RNA from the basal ganglia hematoma was isolated using TRIzol reagent (Invitrogen, Thermo Fisher, MA, USA), in accordance with the manufacturer^’^s protocol. PrimeScript^TM^ RT Master Kit (Takara BioInc, Shiga, Japan) was used to synthesize the complementary deoxyribonucleic acid (cDNA). The Applied Biosystems Quant Studio^TM^ 5 (Thermo Fisher Scientific, Waltham, MA, USA) was used to perform qRT-PCR. β-actin was used as an internal control. At least three samples were analyzed in each group, and each reaction was performed in triplicate. The primer’s sequences are listed in Table 1.

**Table 1:** Primers used in RT-PCR.

Primer sequences (5’-3’)		
Gene	Forward	Reverse
IL-1β	CAACCAACAAGTGATATTC	GATCCACACTCTC
	TCCATG	CAGCTGCA
TNF-α	ATGGCCTCCCTCTCAGTTC	TTGGTGGTTTGCTACGACGTG
CD68	GGACTACATGGCGGTGGAAT	TGGTCACGGTTGCAAGAGAA
Arg-1	CGCCTTTCTCAAAAGGACAG	CCAGCTCTTCATTGGCTTTC
CD206	CAAGGAAGGTTGGCATTTGT	CCTTTCAGTCCTTTGCAAGC
β-Actin	AGGCATTGTGATGGACTCCG	AGCTCAGTAACAGTCCGCCTA

### Immunofluorescence

Mice were anesthetized and perfused with 20 ml ice-cold 0.1 mol l^−1^ phosphate-buffered saline (PBS) *via* the cardiac apex, followed by perfusion with 4% paraformaldehyde (PFA; Zhuang et al., 2020). Whole brains were collected and immersed in 4% PFA overnight and 30% sucrose solutions for 72 h at 4°C. Then, the brain samples were cut into 10 μm coronal slices and fixed on slides. The colon tissues were fixed in 4% PFA overnight and cut into 5 μm thick sections. The tissue sections were washed with PBS and after the processing blocked with 5% BSA and 0.3% Triton X-100. Then, the sections were incubated overnight with the following antibodies: Iba-1 (1:500, Abcam, ab5076), CD68 (1:200, Bioss, bs-6049R), Arg-1 (1:500, Proteintech, 16001-1-AP), and Claudin (1:50, Santa Cruz Biotechnology, sc-166338) at 4°C. After washing with PBS, the cryosections were incubated with Alexa Fluor 488-conjugated donkey anti-rabbit IgG (Invitrogen, A-21206), Alexa Fluor 594-conjugated donkey anti-goat IgG (Invitrogen, A-11058), Alexa Fluor 488-conjugated donkey anti-mouse IgG (Invitrogen, A-21202), Alexa Fluor Plus 594-conjugated donkey anti-mouse IgG (Invitrogen, A-32744), Alexa Fluor 555-conjugated donkey anti-rabbit IgG (Invitrogen, A-31572), Alexa Fluor 488-conjugated donkey anti-goat IgG (Invitrogen, A-32814) and Alexa Fluor 488-conjugated donkey anti-rat IgG (Invitrogen, A-21208) at 37°C for 1 h. Finally, the brain sections were stained with DAPI (Abcam, ab104135), and observed and analyzed under a fluorescence microscope (Leica, Mannheim, Germany). Three sections from each mouse were examined. The mean number of stained cells in each brain section was examined in three fields. The mean fluorescence intensity in each colon section was analyzed using Image J software (NIH).

### Intestinal Permeability Evaluated After ICH

The intestinal permeability following different treatments for ICH in mice, which were fasted for 12 h was assessed using fluorescein isothiocyanate-dextran 4 (600 mg/kg, 100 mg/ml; 4,000 Da FITC-dextran, Sigma). The blood samples were collected after gavage for 4 h and the plasma samples were obtained by centrifugation at 2,500× *g* for 10 min (Kigerl et al., 2016). The fluorescence intensity of undiluted plasma was analyzed using a fluorescence spectrophotometer (SoftMax^®^ Pro5, Molecular Devices) at excitation and emission wavelengths of 485 nm and 535 nm, respectively, and normalized to that of plasma in mice in the sham group and expressed as a percentage of fluorescence per mouse. The researchers were blinded to the experiment during analysis.

### LBP Assay

A mouse LBP ELISA kit was used to determine the level of LBP in the circulation, according to the manufacturer’s instructions (Nanjing Jian Cheng Bioengineering Institute, China). Briefly, the plasma samples were added to the enzyme wells with specific antibodies. Then, a horseradish peroxidase (HRP) recognition antigen was added to each well. After incubation at 37°C for 30 min, both competed with the solid phase antigen and formed an immune complex. The combined HRP-catalyzed tetramethyl benzidine exhibited blue color, and upon interaction with acids, the complex turned yellow. The absorbance at 450 nm was detected using a multifunctional enzymatic instrument (Multimode Plate Reader EnVision, PerkinElmer).

### Antibiotic Depletion of Gut Microbiome in Mice

Wild-type C57 mice were treated with fresh broad-spectrum antibiotics (added in the drinking water daily for 4 weeks and renewed every 3 days, as previously described; Ma et al., 2021). The drinking water contained ampicillin (500 mg l^−1^), metronidazole (500 mg l^−1^), neomycin (500 mg l^−1^), and vancomycin (250 mg l^−1^), abbreviated as AMNV (Sangon Biotech, Shanghai, China). Mice in the vehicle group were treated with an equal volume of drinking water. Antibiotic treatment depleted most of the fecal bacteria; this effect has been confirmed in many studies (Benakis et al., 2020; Hou et al., 2021).

### Fecal Microbiota Transfer (FMT)

FMT was performed as previously reported (Kim et al., 2020; Yu et al., 2021). Briefly, fresh fecal pellets were collected between 9 and 10 am from five ICH mice with ICH treated with Met. The fresh fecal pellets were then diluted in ice-cold PBS (120 mg feces/1 ml PBS). The stool was steeped in the cold PBS for 5 min, followed by homogenization for 10 min, and was finally centrifuged at 1,000*×* g at 4°C for another 10 min. The supernatant was transferred to a new tube and used for the following transplantation. Before ICH induction, the recipient mice were orally administered broad-spectrum antibiotics for 4 weeks. After ICH, 100 μl of the fecal supernatant was gavaged to the recipient mice daily for 14 days. On day 1, the mice in the ICH+FMT group received the fecal supernatant from mice treated with Met for 1 day, and on day 2, the mice received the fecal supernatant from mice treated with Met for 2 days, and so on, for 14 days. Mice in the vehicle group were treated with an equal volume of PBS.

### Gut Microbiota Analysis

The gut microbiota was analyzed using 16S rRNA sequencing (LC-Bio Technology Company, Zhejiang, China). To avoid contamination by exogenous bacteria, fresh feces were collected after abdominal massage. E.Z.N.A.^®^Stool DNA Kit (D4015, Omega, Inc., USA) was used for DNA extraction. The total DNA concentration was measured on a Qubit fluorometer (Thermo Fisher, MA, USA). PCR amplification of the 16S rRNA sequence was performed using a special primer. The final PCR products were purified using AMPure XT beads (Beckman Coulter Genomics, Danvers, MA, USA). The amplicon pools were prepared for sequencing and the size and quantity of the amplicon library were analyzed using the Agilent 2100 Bioanalyzer (Agilent, USA) and the Library Quantification Kit for Illumina (Kapa Biosciences, Woburn, MA, USA). Based on the unique barcodes, paired-end reads were assigned to the samples and truncated by cutting off the barcode and primer sequences. FLASH software was used to merge the paired-end reads., To obtain high-quality clean tags, quality filtering of the raw reads was performed under specific filtering conditions according to the formula. Vsearch software (v2.3.4) was used to filter the Chimeric sequences. After dereplication by DADA2, a feature table and feature sequence were obtained. By random normalization of the same sequences, alpha and beta diversity were calculated. By using the relative abundance of each sample, the feature abundance was normalized according to the SILVA (release 132) classifier. Alpha diversity was applied to analyze the complexity of species diversity for a sample through Chao 1 and Shannon indices which are all calculated using QIIME2. QIIME2 was also used to calculate beta diversity, and the graphs were drawn using the R package. BLAST was used for sequence alignment and the SILVA database was used to annotate the feature sequences for each representative sequence. Other diagrams were drawn using R package (V3.5.2).

### Statistical Analysis

All data are expressed as the mean ± standard error of the mean (SEM). Data which met the normal distribution and homogeneity of variance were analyzed. Multiple groups were compared using the one-way analysis of variance. Two-way repeated-measures ANOVA followed by Tukey’s *post hoc* test was used to analyze the persistent neurological functions. Non-normal distribution and unequal variance parameters were compared by using the Kruskal-Wallis test and Bonferroni test for *post hoc* comparisons. Results were considered statistically significant at *P* < 0.05. Statistical analyses were conducted using GraphPad Prism 8.0 (GraphPad Prism Software Inc, San Diego, CA), and SPSS 22.0 for Window (SPSS, Inc., Chicago, IL).

## Results

### Met Reduces Neuroinflammation by Regulating the Microglia/Macrophage Phenotype After ICH

**Figure 1:**
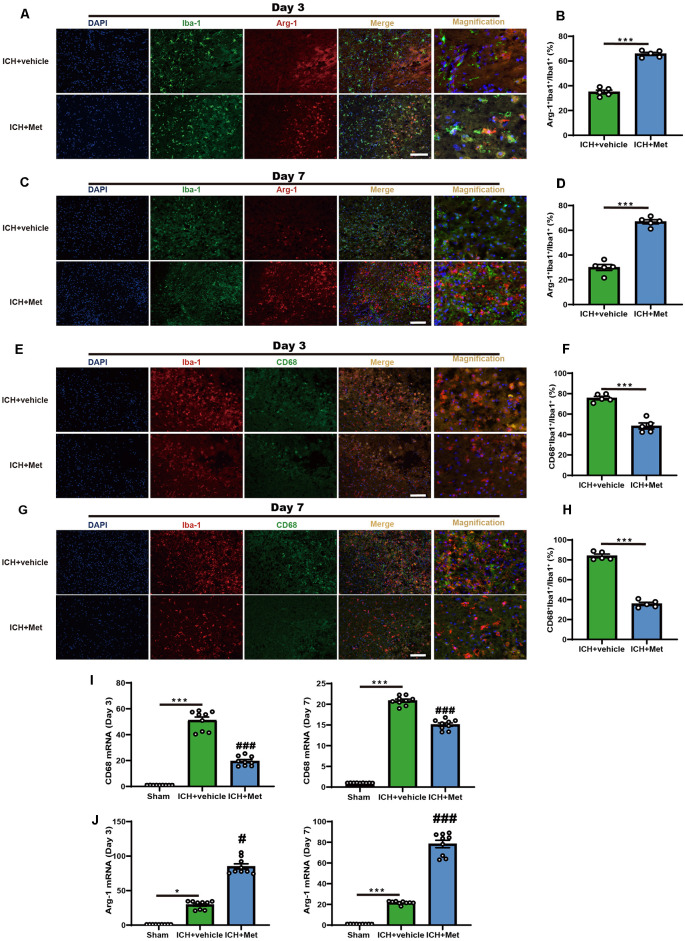
Metformin promoted the phenotype of microglia/macrophage transformed into anti-inflammation and reduced the percentage of pro-inflammatory microglia/macrophage in the peri-hematoma region after ICH. **(A,B)** Immunostaining for Arg-1^+^Iba-1^+^/Iba-1^+^ in ICH + vehicle and ICH + Met group after ICH day 3 (*n* = 5 per group). **(C,D)** Immunostaining for Arg-1^+^Iba-1^+^/Iba-1^+^ in ICH + vehicle and ICH + Met group after ICH day 7 (*n* = 5 per group). **(E,F)** Immunostaining for CD68^+^Iba-1^+^/Iba-1^+^ in ICH + vehicle and ICH + Met group after ICH day 3 (*n* = 5 per group). **(G,H)** Immunostaining for CD68^+^Iba-1^+^/Iba-1^+^ in ICH + vehicle and ICH + Met group after ICH day 7 (*n* = 5 per group). **(I)** The levels of mRNA transcription of CD68 were examined by PCR in sham, ICH + vehicle, and ICH + Met groups after ICH day 3 and 7 (*n* = 3 per group). **(J)** Relative mRNA expression of Arg-1 in sham, ICH + vehicle, and ICH + Met groups after ICH day 3 and 7 (*n* = 3 per group). Data are expressed as the mean ± SEM. *P < 0.05. ***P < 0.001 sham vs. ICH + vehicle group. ^#^*P* < 0.05. ^###^*P* < 0.001 ICH + vehicle vs. ICH + Met group. Scale bar = 100 μm.

We examined the transitions in microglial/macrophage phenotypes in the peri-hematoma region using immunofluorescence to explore the effects of Met on the ICH-induced neuroinflammation. Met treatment significantly increased the Arg-1^+^ Iba-1^+^/Iba-1^+^ cell ratio on days 3 and 7 after ICH induction compared to that in the vehicle group (Figures 1A–D). In contrast, the percentage of CD68^+^ Iba-1^+^/Iba-1^+^ cells were significantly decreased (Figures 1E–H).

In addition, we observed the temporal changes in the expression of inflammatory phenotype signature genes in the brain hemisphere after ICH. The mRNA expression levels of pro-inflammatory markers, such as CD68, IL-1β, and TNF-α, were significantly upregulated at 3 and 7 days after ICH; however, these effects were reversed after treatment with Met (Figure 1I and Supplementary Figures 1A,B,D,E). In contrast, the expression levels of Arg-1 and CD206 were enhanced in the ICH + Met group compared to those in the vehicle group mice (Figure 1J and Supplementary Figures 1C,F). These data suggest that Met reduces neuroinflammation by regulating the microglial/macrophage phenotype following an ICH.

### Met Ameliorates ICH-Induced Brain Injury and Improves Neurological Function

**Figure 2:**
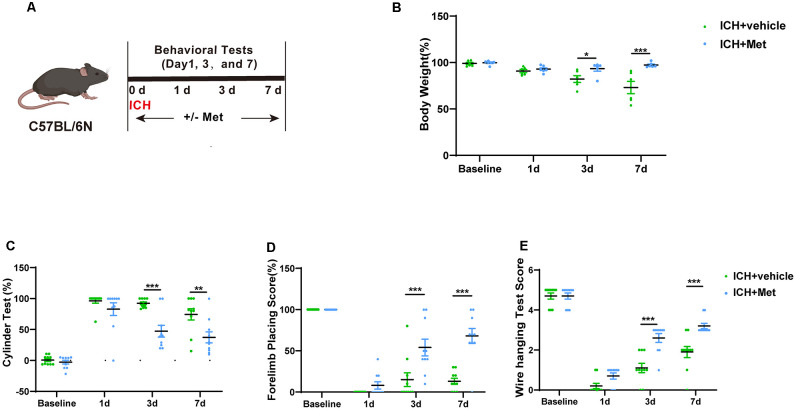
Effects of Metformin on neurological function on mice after ICH. **(A)** Schematic representation of this part experiment. **(B)** The change of body weight in ICH mice after Met treatment at baseline, 1, 3, and 7 days (*n* = 6). **(C–E)** The percentage of cylinder test, forelimb test, and the score of wire hanging test in ICH + vehicle and ICH + Met group (*n* = 10 per group). Data are expressed as the mean ± SEM. *P < 0.05. **P < 0.01. ***P < 0.001 ICH + vehicle vs. ICH + Met group.

Met or vehicle was administered to mice with ICH to investigate the effect of Met treatment on brain injury after ICH (Figure 2A). To do this, we first evaluated the changes in the weight of mice in the vehicle and ICH + Met groups. After ICH, the weight of the mice was reduced from days 1 to 7. In contrast, mice in the ICH + Met group showed weight loss on day 1 but began to recover from days 3 to 7 (Figure 2B).

Next, we performed various neurobehavioral function tests, including the cylinder test, forelimb placing test, and wire-hanging test. The two groups were subjected to the same cylinder and forelimb placing tests before and 1 day after ICH induction. In the cylinder test, mice in the ICH + Met group showed a significant decrease in the positive scores on days 3 and 7 (compared to those in the vehicle group; Figure 2C). In the forelimb placing test, mice in the ICH + Met group showed a significant improvement on days 3 and 7 compared to those in the ICH + vehicle group (Figure 2D). In the wire-hanging test, mice in the vehicle group displayed lower wire-hanging test scores from days 3 to day 7 (Figure 2E).

### Met Treatment Modulates Gut Microbiota After ICH

**Figure 3:**
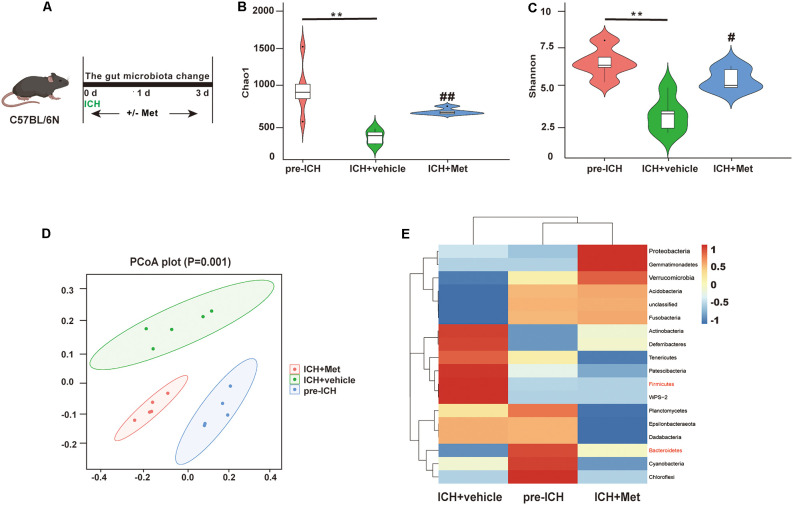
Metformin treatment modulates the community structure of gut microbiota after ICH. **(A)** Schematic diagram of the experiment. **(B,C)** The α-diversity of gut microbiota is represented by Chao1 and Shannon index in pre-ICH, ICH + vehicle, and ICH + Met group mice. **(D)** The β-diversity of gut bacterial was represented by PCoA analysis between three groups. **(E)** The heatmap of most differentially abundant features at the Phylum level of gut microbiota in pre-ICH, ICH + vehicle, and ICH + Met group (*n* = 5 per group). **P < 0.01 pre-ICH vs. ICH + vehicle group. ^#^*P* < 0.05. ^##^*P* < 0.01. ICH + vehicle vs. ICH + Met group.

The gut microbiota plays a key role in ICH-induced neuroinflammation. We determined whether Met administration affected the gut microbiome composition after ICH (Figure 3A). We performed high-throughput 16S rRNA gene sequencing analysis of fecal samples from the ICH + vehicle and ICH + Met groups. First, we assessed α-diversity values of gut microbiota based on Chao1 and Shannon analyses. The results showed that ICH significantly decreased the Chao1 and Shannon indices compared to those in the pre-ICH fecal samples. After Met treatment, the α-diversity value showed significant recovery (Figures 3B,C). The β-diversity value determined by principal coordinate analysis (PCoA) plots showed that the three groups had different gut microbiota community structures (Figure 3D).

A previous study reported that an increased *Firmicutes:Bacteroidetes* ratio was a representation of gut microbiota dysbiosis (Spychala et al., 2018). We compared the relative abundance of the gut microbial flora after Met treatment. At the phylum level, ICH increased the relative abundance of *Firmicutes, Patescibacteria, Tenericutes, Deferribacteria, and Actinobacteria* and decreased *Bacteroidetes, Fusobacteria, Acidobacteria*, and *Verrucomicrobia*. After Met treatment, the composition of the gut microbiota was restored. In addition, the *Firmicutes/Bacteroidetes* ratio was decreased after Met administration (Figure 3E). These data indicate that Met promoted the restoration and maintenance of gut microbiota homeostasis after ICH.

### Met Restores Intestinal Integrity and Reduces the Level of LBP After ICH

**Figure 4:**
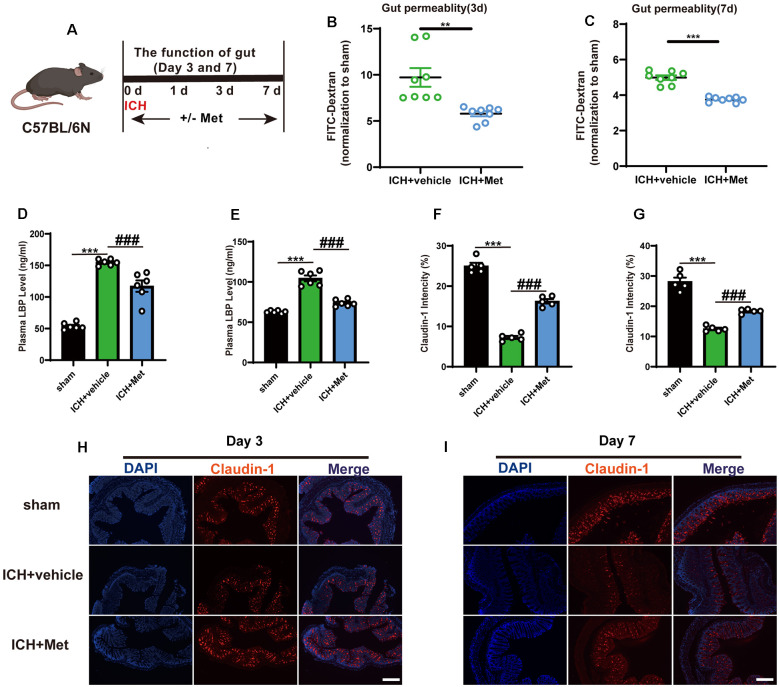
Effect of Metformin on gut permeability and gut intestinal integrity after ICH. **(A)** Experiment design of this part. **(B,C)** Met treatment improved the intestinal permeability after ICH day 3 and 7 (*n* = 4 per group). **(D,E)** The level of LBP in plasma after Met treatment on day 3 and 7 (*n* = 3 per group). **(F,G)** The mean of densities of Claudin-1 after ICH day 3 and 7. **(H,I)** The representative image of immunofluorescence staining of the tight junction protein Claudin-1 in ICH + vehicle and ICH + Met at day 3 and 7 (*n* = 5 per group). **P < 0.01. ***P < 0.001 sham vs. ICH + vehicle group. ^###^*P* < 0.001 ICH + vehicle vs. ICH + Met group.

We previously showed that ICH can increase intestinal permeability in mice, and ICH-induced impairment of gut function may contribute to gut microbiota dysbiosis (Yu et al., 2021). To explore whether Met treatment affected ICH-induced damage of the integrity of the intestinal barrier (Figure 4A), two groups of mice were gavaged with FITC-dextran for 3 and 7 days after ICH, and then the level of FITC-dextran in the plasma was measured. The results showed that Met treatment reversed intestinal permeability barrier damage on days 3 and 7 (Figures 4B,C). Intestinal barrier dysfunction also permits the passage of bacteria and their metabolites, such as LPS, into the systemic circulation, where it binds to the soluble acute-phase protein LBP. Therefore, the level of LBP in the plasma was used as a marker of intestinal permeability. The level of LPB was significantly higher in mice with ICH than in the sham mice on days 3 and 7 after ICH. LBP concentration decreased after treatment with Met (Figures 4D,E).

Tight junction proteins in the intestine have been shown to be associated with barrier integrity. Therefore, we explored the expression of Claudin-1 in the colon after ICH. Compared with that in the sham group, the mean fluorescence intensity of Claudin-1 was reduced after ICH; however, this effect was reversed after the administration of Met from days 3 (Figures 4F,H) through 7 (Figures 4G,I).

### Met Alleviates ICH-Induced Neuroinflammation in a Gut Microbiota-Dependent Manner

**Figure 5:**
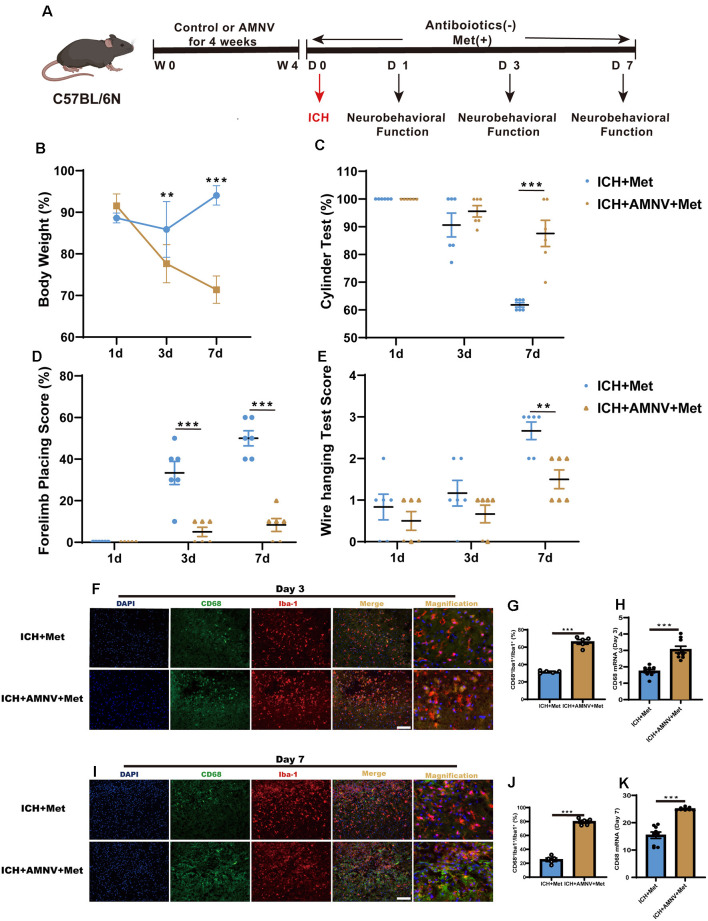
Metformin ameliorated brain injury after ICH in gut microbiota-dependent manner. **(A)** Before ICH, the mice had received the broad-spectrum antibiotic treatment for 4 weeks. **(B)** The change of body weight in ICH + Met and ICH + AMNV + Met mice at baseline, 1st, 3rd, and 7th day (*n* = 6). **(C–E)** Cylinder test, forelimb place test, and the wire hanging test in ICH + Met and ICH + AMNV + Met group (*n* = 6 per group). **(F,G)** Immunostaining for CD68^+^Iba-1^+^/Iba-1^+^ in ICH + Met and ICH + AMNV + Met group after ICH day 3 (*n* = 5 per group). **(H)** PCR examined the levels of mRNA transcription of CD68 in ICH + Met and ICH + AMNV + Met group after ICH day 3 (*n* = 3 per group). **(I,J)** Immunostaining for CD68^+^Iba-1^+^/Iba-1^+^ in ICH + Met and ICH + AMNV + Met group after ICH day 7 (*n* = 5 per group). **(K)** PCR examined the levels of mRNA transcription of CD68 in ICH + Met and ICH + AMNV + Met group after ICH day 7 (*n* = 3 per group). Data are expressed as the mean ± SEM. **P < 0.01. ***P < 0.001 ICH + Met vs. ICH + AMNV + Met group. Scale bar = 100 μm.

We examined whether the intestinal flora was involved in Met regulation, ICH-induced brain injury, and neuroinflammation. Before ICH induction, mice in the ICH + AMNV + Met group were treated with broad-spectrum antibiotics for 4 weeks (Figure 5A). The beneficial effect of Met treatment after ICH was ended in mice in the ICH + AMNV + Met group (Figures 5B–E).

We then explored whether alterations in the intestinal flora due to AMNV affected ICH-induced neuroinflammation. Consistent with our previous observation, Met administration significantly reduced the percentage of CD68^+^Iba-1^+^/Iba-1^+^ cells in the ICH + Met group. However, this effect was reversed in the ICH+ AMNV + Met group after days 3 (Figures 5F,G) and 7 (Figures 5I,J). In addition, the observed increase in the Arg-1 ^+^Iba-1^+^/Iba-1^+^ cell ratio in mice with ICH was reversed after Met treatment (Supplementary Figures 2A–D).

Next, we examined temporal changes in the expression of the inflammatory phenotype signature genes in the brain hemispheres after the depletion of the intestinal flora by AMNV. The decreased mRNA expression levels of the pro-inflammatory marker CD68 in mice with ICH were reversed in the ICH + AMNV + Met group after Met administration (Figures 5H,K). In addition, the expression level of Arg-1 displayed an opposite trend between the ICH + Met and ICH + AMNV + Met groups (Supplementary Figures 2E,F). These data suggest that the gut microbiota may play a key role in improving neurological function and relieving neuroinflammation.

### Frequent Fecal Microbiota Transplantation From Met-Treated Mice With ICH Ameliorates ICH-Induced Brain Injury and Improves Neurological Function

**Figure 6:**
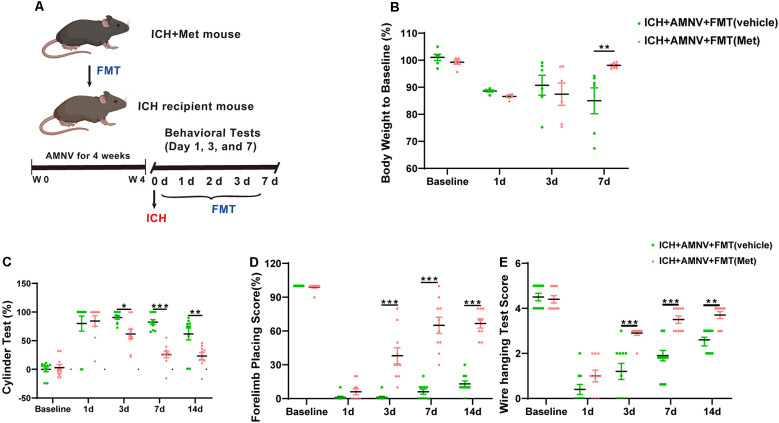
Transplantation of fecal microbiome from metformin treatment ICH mice improves the neurobehavioral function after ICH. **(A)** The detain experiment design of FMT from metformin treatment ICH donor mice to recipient ICH mice. **(B)** Body weight changes in ICH +AMNV + FMT (vehicle) and ICH + AMNV + FMT (Met) group mice at baseline, days 1, 3, and 7 (*n* = 6). **(C–E)** Transplanted ICH mice with the fecal microbiome obtained from metformin treatment ICH donors improved the neurological function as assessed by the cylinder test, forelimb placing test, and wire hanging test after FMT (*n* = 10 per group). Data are expressed as the mean ± SEM. *P < 0.05. **P < 0.01. ***P < 0.001 ICH + +AMNV + FMT (vehicle) vs. ICH + AMNV + FMT (Met) group.

We next attempted to confirm whether the gut microbiota plays an essential role in mediating the therapeutic effect of Met during ICH-induced brain injury. Fecal matter from mice in the ICH + Met and ICH + vehicle groups was transplanted into mice with ICH. The weight and neurobehavioral function between the ICH + AMNV + FMT (vehicle) and the ICH + AMNV + FMT (Met) groups were compared 7 days after FMT (Figure 6A). After ICH induction for 1 day, the weight of the mice was significantly reduced in both groups; and by day 7, the mice in the ICH + AMNV + FMT (Met) group had recovered their weight, whereas those in the ICH + AMNV + FMT (vehicle) group exhibited continued weight loss (Figure 6B).

We also examined the effects of FMT on ICH-induced brain injury. Neurobehavioral function tests were performed as described previously. Both groups showed the same scores in the cylinder and forelimb placing tests pre-ICH induction and at 1 day after ICH onset. In the cylinder test, a significant decrease in positive scores was observed in the ICH + AMNV + FMT (Met) group compared to that in the vehicle group, on days 3, 7, and 14 (Figure 6C). In the forelimb placement test, a significant improvement was observed in the ICH + AMNV + FMT (Met) group compared with the ICH + AMNV + FMT (vehicle) group on days 3, 7, and 14 (Figure 6D). In the wire-hanging test, vehicle group mice displayed lower wire-handing test scores from days 3, 7, and 14 compared with those in the ICH + AMNV + FMT (Met) group (Figure 6E).

### Frequent Fecal Microbiota Transplantation Restores Intestinal Integrity After ICH

**Figure 7:**
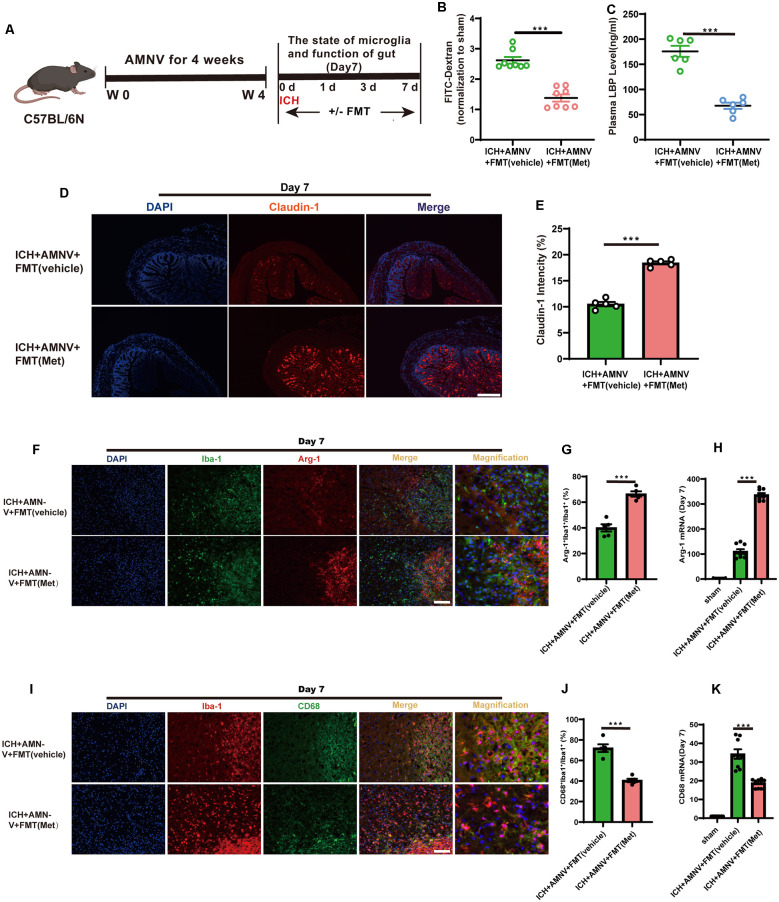
Transplantation of fecal microbiome from metformin treatment ICH mice improves the gut function and alleviates neuroinflammation *via* regulating microglia/macrophage after ICH. **(A)** Experiment design of this part. **(B)** FMT treatment improved the intestinal permeability after ICH day 7 (*n* = 4 per group). **(C)** The level of LBP in plasma after FMT treatment after ICH day 7 (*n* = 6 per group). **(D)** The representative image of immunofluorescence staining of the tight junction protein Claudin-1 in ICH + AMNV + FMT (vehicle) and ICH + AMNV + FMT (Met) at day 7. **(E)** The mean of densities of Claudin-1 after ICH day 7 (*n* = 5 per group). **(F,G)** Immunostaining for Arg-1^+^ Iba-1^+^/Iba-1^+^ in ICH + AMNV +FMT (vehicle) and ICH + AMNV + FMT (Met) group after ICH day 7 (*n* = 5 per group). **(H)** Relative mRNA expression of Arg-1 in ICH + AMNV + FMT (vehicle) and ICH + AMNV + FMT (Met) groups after ICH day 7 (*n* = 3 per group). **(I,J)** Immunostaining for CD68^+^ Iba-1^+^/Iba-1^+^ in ICH + AMNV + FMT (vehicle) and ICH + AMNV + FMT (Met) group after ICH 7 day (*n* = 5 per group). **(K)** Relative mRNA expression of CD68 in ICH + AMNV + FMT (vehicle) and ICH + AMNV + FMT (Met) groups after ICH day 7 (*n* = 3 per group). Data are expressed as the mean ± SEM. ***P < 0.001 ICH + AMNV +FMT (vehicle) vs. ICH + AMNV + FMT (Met) group. Scale bar = 100 μm.

We assessed the regulatory effect of FMT from Met-treated mice on the integrity of the intestinal barrier after ICH (Figure 7A). After FMT treatment for 7 days, the intestinal permeability, plasma LBP concentration, and expression level of tightened junction proteins in the colon were measured. FMT treatment reversed the damage to the intestinal permeability barrier (Figure 7B). The concentration of LBP in plasma of FMT-treated mice was lower than that in mice from the ICH + AMNV + FMT (vehicle) group (Figure 7C). In addition, the mean fluorescence intensity of Claudin-1 increased after FMT (Met) treatment compared to that in the ICH + AMNV + FMT (vehicle) group (Figures 7D,E). These data demonstrated that FMT from Met treatment mice reduced the gut permeability.

### Frequent Fecal Microbiota Transplantation Ameliorates Neuroinflammation by Regulating Microglia/Macrophage Phenotype After ICH

We explored the effects of FMT on the changes in the microglial/macrophage phenotypes in the peri-hematoma region. The results demonstrated that FMT from Met-treated mice significantly increased the Arg-1^+^Iba-1^+^/Iba-1^+^ cell ratio after day 7 of ICH induction compared to in the FMT from mice in the vehicle group (Figures 7F,G); in contrast, the percentage of CD68^+^Iba-1^+^/Iba-1^+^ cells was significantly decreased (Figures 7I,J).

In addition, we examined the temporal changes in the expression of inflammatory phenotype signature genes in the brain of mice with ICH following FMT treatment. The expression of Arg-1 was enhanced in the ICH + AMNV + FMT (Met) group compared to that in the vehicle group (Figure 7H). The transcript-level expression of the pro-inflammatory marker CD68 was upregulated at day 7 after ICH; however, this effect was reversed after treatment with FMT from Met-treated mice (Figure 7K). These data suggest that FMT from Met-treated mice reduced neuroinflammation by regulating the microglial/macrophage phenotype following ICH. Therefore, the gut microbiota plays a key role in mediating the therapeutic effect of Met during ICH-induced brain injury.

## Discussion

In this study, we showed that Met regulates ICH-induced neuroinflammation by modulating the microglial/macrophage phenotype in the brains of mice with ICH. We further show that Met mediates these therapeutic effects by modulating the gut microbiota composition and promoting neurofunctional recovery after ICH in mice. In addition, this study showed that Met treatment restored intestinal integrity and reduced LBP levels after ICH.

Previous studies suggested that ICH triggers a robust neuroinflammatory response (Fu et al., 2021a). Microglia/macrophages can be rapidly activated after ICH and play a key role in mediating the neuroinflammatory response (Li et al., 2017; Zhu et al., 2019). After activation, the microglial/macrophage phenotype is simplified as either a pro- or anti-inflammatory phenotype (Bai et al., 2020). Studies have suggested that changing the phenotype of microglia/macrophage from a pro- to an anti-inflammatory state had a neuroprotective effect in ICH animal models (Lan et al., 2017; Chen et al., 2020; Fu et al., 2021a, b). However, research has mainly focused on the local inflammatory immune response in the brain. Unfortunately, most of those findings have not been translated to the clinical setting, and the prognosis of patients with ICH remains poor. Therefore, more effort is needed to explore the potential targets for ICH therapy. Recently, the focus of studies has shifted to the regulation of peripheral and systemic immune responses in the CNS after ICH (Li et al., 2020; Shi et al., 2021). However, the role of peripheral and systemic immune response in ICH remains unknown.

Numerous studies have shown that the gut microbiota is involved in the regulation of inflammatory responses in many CNS diseases (Mueller et al., 2021). In a mouse model of TBI, the gut microbiota plays an essential role in neuroinflammation, neurofunction, and neurogenesis (Celorrio et al., 2021). SCI disrupts the microbiome and restoration of the gut microbiota homeostasis *via* FMT and improves locomotor and intestinal function (Jing et al., 2021). In addition, we previously reported that ICH can induce microbiota dysbiosis, which, in turn, can contribute to ICH-induced neuroinflammation in the brain (Yu et al., 2021). However, the correlation between neuroinflammation and the gut microbiota after ICH has not been widely examined. Therefore, understanding the regulation of the gut microbiota may provide new insights for ICH treatment.

Met is the most commonly used therapeutic agent for diabetes mellitus, and many studies have indicated that Met also exerts a treatment effect on CNS diseases. Treatment with Met exerted anti-inflammatory effects and prevented brain injury after IS (Liu et al., 2014; Zemgulyte et al., 2021). In Parkinson’s disease (PD) mice models, Met reduced mitochondrial respiration and inhibited neuroinflammation, which significantly improved motor function and neuronal viability (Mor et al., 2020; Ryu et al., 2020). In this study, we hypothesized that Met is an essential regulator of neuroinflammation after ICH. Our results indicated that the administration of Met after ICH significantly attenuated neuroinflammatory responses by shifting the phenotype of microglia/macrophage toward the anti-inflammatory response. These results were consistent with decreased expression levels of pro-inflammatory markers in the microglia/macrophage. Furthermore, Met treatment increased the expression levels of anti-inflammatory microglial/macrophage markers and significantly alleviated neuroinflammation after ICH, thereby contributing to the recovery of neurological function.

Given that Met is widely prescribed in clinical practice, our results suggest a significant clinical value for patients with ICH. In previous studies, the mechanism of neuroprotection by Met was found to be mainly dependent on the AMPK signaling pathway (Liu et al., 2014; Hill et al., 2016). In a recent study, Ma et al. (2021) reported that Met reshapes the gut microbiota composition and inhibits microglial/macrophage activation and neuroinflammation in the brains of obese mice. The anti-inflammatory effects of Met after ICH have also been reported (Qi et al., 2017). Consequently, whether Met exerts therapeutic effects by regulating ICH-induced neuroinflammation through the gut microbiota composition is unclear. Therefore, the relationship between Met and the gut microbiota in the regulation of neuroinflammation after brain injury should be explored in further detail. Here, we observed that Met regulates the gut microbiota composition and promotes gut microbiota homeostasis in mice with ICH. In addition, depleting the mice intestinal flora of mice with broad-spectrum antibiotics blocked the anti-inflammatory and neuroprotective effects of Met after ICH induction. Intragastric administration of feces from Met-treated mice with ICH attenuated ICH-induced neuroinflammation. This result was in agreement with the decreased expression levels of pro-inflammatory markers in the microglia/macrophages. In addition, treatment with FMT from Met-treated mice significantly promoted the recovery of neurological function. These results suggest a possible mechanism by which Met treatment restores gut microbiota homeostasis following ICH and attenuates the neuroinflammatory responses to improve neurological function. However, the underlying mechanism underlying the effect of Met on the gut microbiota composition after ICH requires further analysis.

Previous studies have suggested that after acute brain injuries patients are often afflicted with intestinal dysfunction complications (Olsen et al., 2013; Singh et al., 2016; Cheng et al., 2018). We previously also showed that ICH induces intestinal paralysis and increased intestinal permeability in mice, and demonstrated that gut dysfunction is a cause of gut microbiota dysbiosis. Ahmadi et al. (2020) found that Met alleviates gut permeability and aging-induced inflammation. Next, we explored whether Met could regulate gut function after ICH. Met treatment improved gut epithelial permeability and reversed the expression of epithelial tight junction proteins after ICH in mice. Met treatment also decreased the level of plasma LBP, which can enter the circulatory system during ICH when the gut barrier is impaired. In addition, treatment with FMT from Met-treated mice significantly promoted the recovery of gut barrier function, which increased the expression of epithelial tight junction proteins and reduced the levels of FITC-dextran and plasma LBP. These results suggest that Met can rescue gut function after ICH and that the gut microbiota plays a crucial role in this process. However, the underlying mechanisms should be further examined.

There were several limitations to this study. First, we did not determine the mechanism underlying the changes in gut microbiota after Met administration during ICH-induced neuroinflammation response. Therefore, further research is required to explore the association between intestinal microbiota and neuroinflammation. Second, we investigated the effects of the gut flora in male mice. However, estrogen levels and sex may affect ICH outcomes (Chang et al., 2020). Therefore, further studies are necessary to investigate the effect of Met and the gut microbiota in female mice with ICH. Third, age is an important factor that affects the composition of the gut microbiota and the functional outcomes of many CNS diseases (Spychala et al., 2018; Lee et al., 2020). However, we only focused on the intestinal microbiota in young mice; therefore, it will be necessary to explore the function and underlying mechanism of the gut microbiota in older ICH mouse models. Fourth, the peripheral cells that infiltrated the brain after ICH play a key role in ICH-induced neuroinflammation. In future studies, we will explore the effect of Met treatment on the infiltration of peripheral cells, including the monocytes, T cells, neutrophils and macrophage, into the brain during ICH. Lastly, rapid gut microbiome dysbiosis can exacerbate brain injury after IS (Xu et al., 2021). Since IS and ICH display similar pathological processes, we postulate that rapid gut microbiota changes also play a key role in ICH. Our future work will explore the effect of Met on gut microbiota dysbiosis in hyperacute ICH.

## Conclusion

Our results demonstrated that Met alters the gut microbiota composition in mice with ICH, a phenomenon that may alleviate neuroinflammation by regulating the microglial/macrophage phenotype and improving neurological function. Our findings may provide new insights into the prevention of ICH-induced secondary brain injury.

## Data Availability Statement

The datasets presented in this study can be found in online repositories. The names of the repository/repositories and accession number(s) can be found below: https://www.ncbi.nlm.nih.gov/ PRJNA779790.

## Ethics Statement

The animal study and all experimental procedures were reviewed approved and supervised by the Institutional Ethics Committee of the Second Affiliated Hospital, Medicine School of Zhejiang University.

## Author Contributions

GC, FL, and LW conceived and designed the study. XF, XY, and XW performed the ICH model and PCR. HZ, LH, and GZ performed the immunostaining. XF and CX prepared the figures. JLiu modified the figures. XY and JLi analyzed data. XF, GZ, and SC prepared the manuscript draft. GC, FL, and LW wrote the article. All authors contributed to the article and approved the submitted version.

## Conflict of Interest

The authors declare that the research was conducted in the absence of any commercial or financial relationships that could be construed as a potential conflict of interest.

## Publisher’s Note

All claims expressed in this article are solely those of the authors and do not necessarily represent those of their affiliated organizations, or those of the publisher, the editors and the reviewers. Any product that may be evaluated in this article, or claim that may be made by its manufacturer, is not guaranteed or endorsed by the publisher.
