# Supplementary material for: Metformin Alleviates Neuroinflammation Following Intracerebral Hemorrhage in Mice by Regulating Microglia/Macrophage Phenotype in a Gut Microbiota-Dependent Manner
Source: Front Cell Neurosci. 2022 Jan 18;15:789471. doi: 10.3389/fncel.2021.789471 (PMC8806158; doi:10.3389/fncel.2021.789471)
Supplement: Supplementary file 1 [file Data_Sheet_1.docx]

**Supplementary Material**


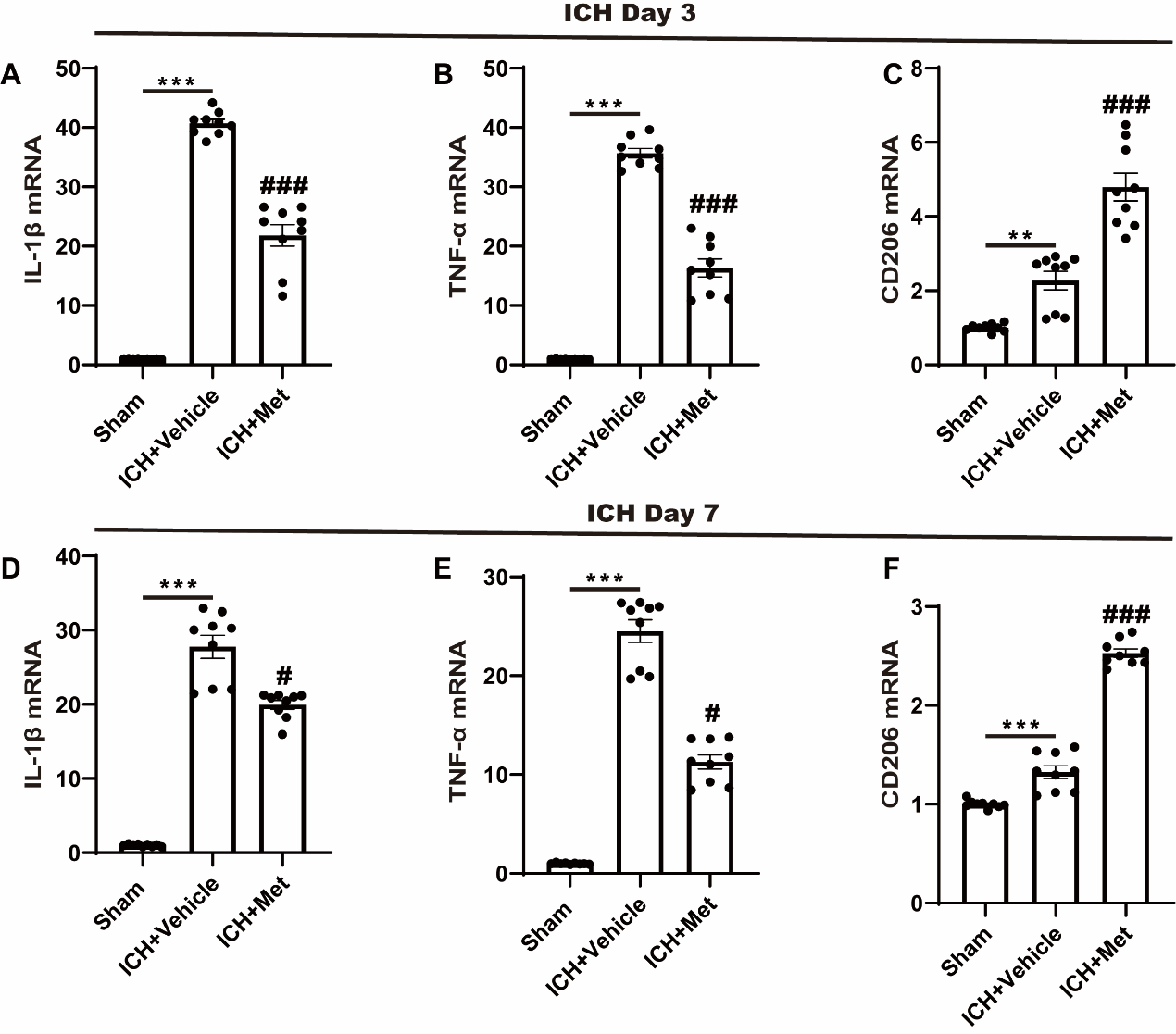


**Supplement Figure 1.** The levels of mRNA transcription of IL-1β, TNF-α, and CD206 were examined by PCR in sham, ICH + Vehicle, and ICH + Met groups after ICH day 3 (**A-C**) and day 7 (**D-F**). (n = 3 per group).


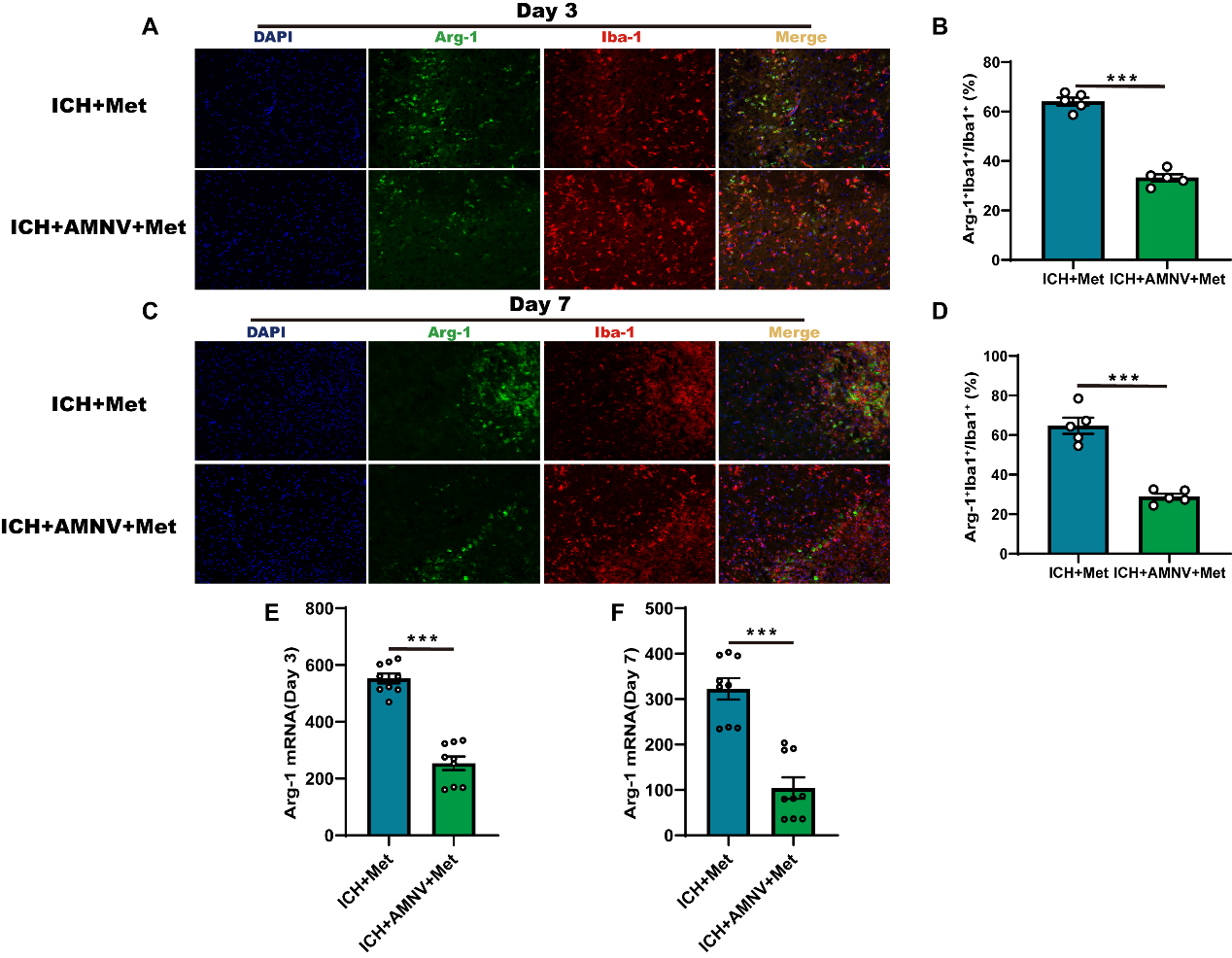


**Supplement Figure 2.** **A and B.** Immunostaining for Arg-1^+^Iba-1^+^/Iba-1^+^ in ICH + Met and ICH + AMNV + Met group after ICH 3 day. (n = 5 per group). **C and D.** Immunostaining for Arg-1^+^Iba-1^+^/Iba-1^+^ in ICH + Met and ICH + AMNV + Met group after ICH 7 day. (n = 5 per group). **E**. The levels of mRNA transcription of Arg-1 in ICH + Met and ICH + AMNV + Met group after ICH 3 day. (n = 3 per group). **F.** The levels of mRNA transcription of Arg-1in ICH + Met and ICH + AMNV + Met group after ICH 7 day. (n = 3 per group). Data are expressed as the mean ± SEM. **P*<0.05. ***P*<0.01. ****P*<0.001 ICH + Met vs. ICH + AMNV + Met group. Scale bar = 100μm.

**Table S1. Study groups and animal used**

| Experiment 1 | Numbers | Dead |
| --- | --- | --- |
| Groups |  |  |
| ICH+Vehicle | 10 |  |
| ICH+Met | 10 |  |
| Experiment 2 |  |  |
| Groups |  |  |
| Sham d3  Sham d7 | 3  3 |  |
| ICH d3+Vehicle | 8 |  |
| ICH d7+Vehicle | 8+1 | 1 |
| ICH d3+Met | 8 |  |
| ICH d7+Met | 8 |  |
| Experiment 3 |  |  |
| Groups |  |  |
| Sham d3  Sham d7 | 15  15 |  |
| ICH d3+Vehicle | 15 |  |
| ICH d7+Vehicle | 15+1 | 1 |
| ICH d3+Met | 15 |  |
| ICH d7+Met | 15 |  |
| Experiment 4 |  |  |
| Groups |  |  |
| ICH+Vehicle | 5 |  |
| ICH+Met | 5 |  |
| Experiment 5 |  |  |
| Groups |  |  |
| Sham | 6 |  |
| ICH+Met | 14 |  |
| ICH+AMNV+Met | 14+1 | 1 |
| Experiment 6 |  |  |
| Groups |  |  |
| Sham | 13 |  |
| ICH+Vehicle | 33+1 | 1 |
| ICH+FMT | 33 |  |
| Total | 275 | 4 |
| Mortality |  | 1.45% |
